# Inversion of the Uterus

**Published:** 1860-12

**Authors:** Ira Hatch


### INVERSION OF THE UTERUS.

Case reported to the Chicago Medical Society,

BY IRA HATCH, M. D.

(Published by Request.)

Mrs. L. H——, of Downer's Grove, Dupage Co., was taken in labor with her second child Sept. 16th, 1860. Dr. Brown, of Danby Station, a physician of intelligence and experience, was in attendance. He says that the labor progressed slowly but favorably. The presentation was natural, and everything promised a favorable termination; although the pains were short and light, and the contractions of the uterus were feeble, still owing to the relaxed condition of the parts, the head of the child continued to advance. During an examination, the membranes were ruptured, and immediately a strong and continued pain came on which expelled the child with great force. Considerable hemorrhage flowed, and some time elapsed before any contraction of the uterus took place. Frictions to the abdomen, and the usual means for exciting contraction were resorted to, but no efforts were made, such as pulling at the cord to remove the placenta. While the Doctor was quietly waiting, suddenly, without the least warning the patient was seized with a violent throe, and instantly almost the uterus with the placenta attached to the fundus was expelled, being completely inverted. This was succeeded by profuse flooding and fainting fits rapidly following each other. The Doctor quickly detached the placenta and deposited the uterus in the vagina, after which the flowing in a great measure ceased. Great exhaustion continued for several hours. This precluded all efforts at re-inversion at this time. She rallied slowly under the use of opium and stimulants, and continued to gain strength until the 18th, three days after the accident, when I saw her.

I found her pale and feeble, with a frequent pulse, but quiet, comfortable and cheerful. There was no pain or hemorrhage, and it was thought that she would bear the operation of replacing the uterus. On examination I found it occupying the entire vagina, the fundus presenting just within the vulva. It seemed to have no disposition to descend any lower after it was placed there. By following with the finger the *cul de sac* formed by the union of the vagina and uterus, I could trace the neck entirely around. Not a trace of the os tincæ could be found, showing most conclusively that the inversion was complete. The surface where the placenta had been attached was rough, and had a feeling similar to the corresponding portion of the placenta, and was not very sensative. But the remaining portion of the surface was smooth and very sensative, particularly around the neck. There was a slight depression at the center of the fundus which may be taken as a diagnostic mark of an inverted uterus. This depression could be easily increased by pressing, giving a cup-like form to the fundus. Pressure at this point seemed to be borne well; and did not seem to excite the organ; while manipulation about the neck, or pressing upon the body, the neck would excite pain and bearing down. It was therefore agreed to attempt the reduction by unfolding the fundus and pushing its center up through the neck, and not by attempting to unfold from the neck. It gave great pain when the organ was grasped and pressed upwards, but far less when the fundus was indented and carried upwards. No attempt was made at this time to reduce the organ, as we were satisfied that it could not be done without the aid of some suitable instrument. For the os and neck were pretty well contracted by this time, and for the want of an instrument it was postponed till the next day, four days after the accident happened. As the patient was gaining strength every day we felt in no hurry. The instrument selected was a rectal bougic abont an inch in diameter and twelve inches in length, sufficiently flexible to give the point the right direction. The body of the womb was as large as the largest sized orange, and very firm and unyielding except at the center of the fundus.

It was resolved to push this up in the shape of a cone, and thus dilate the neck gradually, imitating the action of the membranes in natural labor.

It was resolved to persevere according to this plan alone until success was attained, providing the strength of the patient held out. She was placed upon her back with her hips elevated, and brought partially under the influence of chloroform. The hand being introduced, firm pressure was made upon the center of the fundus with the ball of the thumb, until a cup-like depression was made, sufficient to hold the tube without slipping. The instrument was then passed up by the left hand, and the end was placed in the depression made by the thumb; and being grasped by the fingers, upward pressure was made simultaneously with the tube and the hand. It required a long time to dilate the neck sufficiently to let the tube and the tips of the fingers through the os uteri. As soon as this was accomplished, the tube was withdrawn and the hand was carried up boldly, untill the organ was completely unfolded and contracted strongly around the hand.

The reduction being effected, a vulcanized caoutchouc bag was placed in the vagina, and distended, in order to prevent a return of the malady.

During the operation the patient lost but little blood, and although it was tedious and painful, she bore it with great fortitude, and was less exhausted than might have been expected. The after-pains were somewhat severe, but were soon quieted by anodynes, and she was as comfortable the next morning as she was before the operation. I saw her four days afterward, when she seemed to be improving without any unfavorable symptoms.

Nothing except the continued frequency of the pulse and the offensiveness of the lochia, excited the least apprehension of danger; and these were not so marked in their character as to excite alarm.

She bore her anodynes and tonics well. The stomach and bowels were quiet. She had some appetite for food, and was neither restless, feverish, or irritable. Her pulse was frequent, but as it was soft and regular, the frequency was attributed to the debility consequent upon the loss of blood. The secretion of milk had taken place, and she was able to take her child upon the arm and nourish it without any assistance. There was no peritoneal inflammation, and no fullness or tenderness of the abdomen. The uterus could be felt above the pubes and bore firm pressure without pain. There was no more tenderness of the parts than is usual after natural labor. She continued to improve until the 28th, when a dose of castor oil was given, as the bowels had not moved for five days. It operated actively; a diarrhœa set in, followed by vomiting. She sunk from exhaustion, and died October 1st, sixteen days after her confinement. She was considered convalescent by physician and friends, until the fatal diarrhœa set in. Whether the diarrhœa was the effect of the cathartic, or of some other latent cause, is not easy to determine. That her death was not occasioned by the reduction of the uterus, although the operation was difficult and painful, is evident from the whole history of the case.

This case assumes considerable importance from the fact that it furnishes another instance of *spontaneous inversion*. That it was not occasioned by pulling at the cord to deliver the placenta, we have the positive assertions of Dr. Brown, the attending physician, in whose skill, candor and truthfulness I have the utmost confidence. Much credit is due to him for the prompt manner in which he treated the case, as well as success of the operation of reinversion.

As it is a mooted point whether inversion of the uterus ever takes place spontaneously, no pains were spared in the investigation of the case. The bystanders were questioned on the subject. The mother and aunt of the young lady stood by the bed side at the time. They informed me that she lay perfectly quiet and free from pain. They were talking and laughing as usual on such occasions. Now, every experienced physician knows that pulling upon the cord while the placenta is adherent, will occasion pain. But in this case the first warning was a loud cry from the patient, accompanied by a violent expulsive effort, and all was over. She had fainted. The doctor called for a light and found the uterus completely inverted with the placenta adhering to the fundus, lying between the thighs.

The *rapid termination* of the labor ; the *relaxation* of the parts, and the attachment of the placenta to the *fundus*, were the predisposing causes of the inversion.

